# Patterns of response to aripiprazole, lithium, haloperidol, and placebo across factor scores of mania

**DOI:** 10.1186/s40345-015-0026-0

**Published:** 2015-05-05

**Authors:** Michael J Ostacher, Trisha Suppes, Alan C Swann, James M Eudicone, Wally Landsberg, Ross A Baker, Berit X Carlson

**Affiliations:** VA Palo Alto Health Care System, 3801 Miranda Avenue, Mail Code 151 T, Building 4, 3rd Floor, Palo Alto, CA 94304 USA; Stanford University School of Medicine, Stanford, CA USA; Department of Psychiatry and Behavioral Sciences, Baylor College of Medicine, 1977 Butler Blvd Suite E4.400, Houston, TX 77030 USA; Astrazeneca, 601 Office Center Drive Suite 200, Fort Washington, PA 19034 USA; Otsuka Pharmaceutical Europe Ltd., Gallions – 1st Floor Wexham Springs Framewood Road, London, Wexham SL3 6PJ UK; Otsuka Pharmaceutical Development & Commercialization, Inc., 508 Carnegie Center, Princeton, NJ 08540 USA; Bristol-Myers Squibb, P.O. Box 5200, Princeton, NJ 08543-5200 USA

**Keywords:** Aripiprazole, Bipolar disorder, Treatment outcome, Antipsychotic, Factor analysis, Mania

## Abstract

**Background:**

A previous factor analysis of Young Mania Rating Scale and Montgomery-Åsberg Depression Rating Scale items identified composite factors of depression, mania, sleep disturbance, judgment/impulsivity, and irritability/hostility as major components of psychiatric symptoms in acute mania or mixed episodes in a series of trials of antipsychotics. However, it is unknown whether these factors predict treatment outcome.

**Methods:**

Data from six double-blind, randomized, controlled clinical trials with aripiprazole in acute manic or mixed episodes in adults with bipolar I disorder were pooled for this analysis and the previously identified factors were examined for their value in predicting treatment outcome. Treatment efficacy was assessed for aripiprazole (*n* = 1,001), haloperidol (*n* = 324), lithium (*n* = 155), and placebo (*n* = 694) at baseline, days 4, 7, and 10, and then weekly to study end. Mean change in factor scores from baseline to week 3 was assessed by receiver operating characteristics curves for percentage factor change at day 4 and week 1.

**Results:**

Subjects receiving aripiprazole, haloperidol, and lithium significantly improved mania factor scores versus placebo. Factors most predictive of endpoint efficacy for aripiprazole were judgment/impulsivity at day 4 and mania at week 1. Optimal factor score improvement for outcome prediction was approximately 40% to 50%. Early efficacy predicted treatment outcome across all factors; however, response at week 1 was a better predictor than response at day 4.

**Conclusions:**

This analysis confirms clinical benefits in early treatment/assessment for subjects with bipolar mania and suggests that certain symptom factors in mixed or manic episodes may be most predictive of treatment response.

## Background

Bipolar disorder has historically been described as discrete episodes of depression and mania. However, the structure of an episode is far more complex, and it has long been recognized that depressive and manic symptoms often exist simultaneously during periods of illness and can combine to create so-called ‘mixed states’ (Kraepelin 1921). Subsequent enquiries focused on mixed mania consisting of depressive symptoms during manic episodes (Kotin and Goodwin 1972; McElroy et al. 1992) or defined mixed states as a form of mania combining syndromal depression and mania (Swann et al. 2009). To date, there has been little consensus over the structure and definition of a mixed state, making a correct clinical diagnosis increasingly difficult. There is increasing evidence that depressive features may be a component across episodes of bipolar disorder (APA 2013; Swann et al. 2013a), leading to a new use of the concept of a ‘mixed-features specifier’ in the Diagnostic and Statistical Manual of Mental Disorders, Fifth Edition (DSM-V) (APA 2013) rather than the previous term of mixed episodes as described in the DSM-IV, text revision (APA 2000). It remains to be established, however, whether depression is a characteristic of manic episodes (mania is a characteristic of depressive episodes) or whether ‘mixety’ is a combination of independent depressive and manic states. Better understanding of the clinical structure of manic episodes in subjects with bipolar disorder may be useful in understanding treatment response and determining differences in efficacy based on symptom dimensions.

Several factor analyses exploring the symptomatic structure of manic episodes have confirmed the heterogeneity of manic and mixed episodes (Azorin et al. 2008; Bertschy et al. 2007; Cassidy et al. 1998; Dilsaver et al. 1999; Harvey et al. 2008; Lipkovich et al. 2008; Rossi et al. 2001; Sato et al. 2002; Swann et al. 2001), which has an impact not only on classification of bipolar disorder but also on the choice of treatment. Recent treatment algorithms sought to distinguish between euphoric (classical) mania, dysphoric mania and mixed states, psychotic mania, and hypomania to better personalize treatment (Grunze et al. 2009). Therefore, the symptomatic structure of bipolar disorder is of interest as it can potentially be used to differentially predict response to treatment and guide treatment choice.

One important aspect of treatment strategies for mania is the need for early clinical prediction of response to a particular treatment. Previous analyses showed that early response to lithium or ziprasidone predicted treatment outcome in acute manic and mixed episodes (Ketter et al. 2010; Swann et al. 1986). However, this topic has never been investigated using specific, clinically observable symptoms of manic or mixed episodes. Therefore, using the factor structure developed previously (Swann et al. 2013b), the relationship between early response and treatment outcome was investigated. The analysis was based on a series of randomized clinical trials that led to the approval of aripiprazole for acute manic and mixed states by the Food and Drug Administration (FDA): three double-blind, placebo-controlled, 3-week studies (El Mallakh et al. 2010; Keck et al. 2003; Sachs et al. 2006b), a double-blind, haloperidol-controlled, 12-week study (Vieta et al. 2005), and two active- and placebo-controlled, double-blind, 12-week studies (Keck et al. 2009; Young et al. 2009). Previously, baseline data from these combined studies was used to conduct a rotated factor analysis followed by a cluster analysis using all items from the Young Mania Rating Scale (YMRS) and the Montgomery-Åsberg Depression Rating Scale (MADRS) (Swann et al. 2013b) and to identify the factor structure of psychiatric symptoms of the subjects included in these studies. Subjects were stratified by symptom state according to the DSM-IV criteria. The analysis identified five factors, characterized (in order of variance accounted for) as depression, mania, sleep disturbance, judgment/impulsivity, and irritability/hostility (Swann et al. 2013b). All manic episodes, whether mixed or non-mixed, shared the same factor and cluster structure, differing only in factor scores.

Lithium and haloperidol were chosen as comparators in the included studies because of their proven, evidenced-based use in the treatment of mania. Traditionally, lithium was the major treatment used to ameliorate symptoms of mania by reducing excitatory neurotransmitters (dopamine and glutamate) and is also known for preventing recurrence of manic and depressive episodes (Geddes et al. 2010; Malhi et al. 2013) and reducing the risk of suicide (Tondo and Baldessarini 2000; Cipriani et al. 2013). Haloperidol, a typical antipsychotic, is non-selective and binds to a broad range of receptors, exerting efficacy mainly through the antagonism of dopamine receptors. It is well established as a treatment for acute mania (Cipriani et al. 2011). Aripiprazole is a second-generation atypical antipsychotic with a mechanism of action that differs from other typical and atypical antipsychotics. Aripiprazole is a high-affinity partial agonist of the dopamine D2/D3 receptor, with partial agonist activity at the 5HT1A receptor and antagonist activity at the 5HT2A receptor (Burris et al. 2002; Stark et al. 2007, Tadori et al. 2008). Like lithium, there is evidence that the use of aripiprazole is effective in preventing manic episodes (Goodwin et al. 2011).

The objectives of the *post hoc* analysis presented here are to use these previously determined factors to characterize the effects of aripiprazole, comparative treatment (haloperidol and lithium), and placebo in the management of bipolar I disorder and to assess the value of early efficacy in predicting efficacy at endpoint (week 3).

## Methods

### Study design

This was a *post hoc* analysis of pooled data from six double-blind, randomized, controlled clinical trials with aripiprazole, which included 2,179 subjects (≥18 years) diagnosed with bipolar I disorder, as defined by the DSM-IV (APA 2000), who were experiencing an acute manic or mixed episode (El Mallakh et al. 2010; Keck et al. 2003; Keck et al. 2009; Sachs et al. 2006b; Vieta et al. 2005; Young et al. 2009) (Table 1). The study protocols, procedures, and consent statements were approved by the Institutional Review Boards (IRBs) of each participating site. Details of the study designs, duration, and treatments are given in Table 1. In all six studies, efficacy assessments included the YMRS total score (primary efficacy outcome) and the MADRS Total score. Factor analysis using YMRS and MADRS line items performed using the principal components method with varimax rotation (Tabachnik and Fidell 2007) identified five factors with eigenvalues >1 (Table 2). These factors were depression (factor 1), mania (factor 2), sleep disturbance (factor 3), judgment/impulsivity (factor 4), and irritability/hostility (factor 5) (Swann et al. 2013b).

**Table 1:** **Studies used in factor analysis**

**Study**	**Duration** **(weeks)**	**Efficacy assessments (time points)**	**Study design**	**Entry criteria**		**Treatments**		
				**YMRS total score**	**MADRS** **total score**			
CN138-007 (Sachs et al. 2006a)	3	Baseline Days 2, 4, 7, 10, 14, 21	International, multicenter, double-blind, placebo-controlled study in subjects with bipolar I disorder experiencing acute manic or mixed episodes	≥20 at randomization	–	Aripiprazole (flexibly dosed 15 to 30 mg/day)	Placebo	
CN138-009 (Keck et al. 2003)	3	Baseline Days 4, 7, 10, 14, 21			–	Aripiprazole (flexibly dosed 15 to 30 mg/day)	Placebo	
CN138-074 (El Mallakh et al. 2010)	3	Baseline Days 7, 10, 14, 21			–	Aripiprazole (fixed dose 15 or 30 mg/day)	Placebo	
CN138-008 (Vieta et al. 2005)	12	Baseline Days 4, 7, 10 Weeks 2, 3, 4, 5, 6, 8, 10, 12	International, multicenter, double-blind, active-controlled study in subjects with bipolar I disorder experiencing acute manic or mixed episodes	≥20	–	Aripiprazole (flexibly dosed 15 to 30 mg/day)	Haloperidol (5 to 15 mg/day)	
CN138-135 (Keck et al. 2009)	12	Baseline Days 2, 4, 7 Weeks 2, 3, 4, 5, 6, 8, 10, 12	International, multicenter, double-blind, active- and placebo-controlled study in subjects with bipolar I disorder experiencing acute manic or mixed episodes	≥20 at screening and baseline with <25% decrease between visits	≤17 at screening and baseline with <4-point increase between visits	Aripiprazole (flexibly dosed 15 to 30 mg/day)	Placebo (to week 3 only)	Lithium (900 to 1,500 mg/day)
CN138-162 (Young et al. 2009)	12	Baseline Days 2, 4, 7, 10 Weeks 2, 3, 4, 5, 6, 8, 10, 12				Aripiprazole (flexibly dosed 15 to 30 mg/day)	Placebo (to week 3 only)	Haloperidol (5 to 15 mg/day)

MADRS, Montgomery-Åsberg Depression Rating Scale; YMRS, Young Mania Rating Scale.

**Table 2:** **Factors of bipolar mania identified from aripiprazole studies (Swann et al.** 2013b **)**

**Factors**	**Scale**	**Item**
**(Total % variance)**		
Factor 1: depression (21.3%)	MADRS	Reported sadness
	MADRS	Apparent sadness
	MADRS	Inner tension
	MADRS	Reduced appetite
	MADRS	Lassitude
	MADRS	Inability to feel
	MADRS	Pessimistic thoughts
	MADRS	Suicidal thoughts
Factor 2: mania (12.0%)	MADRS	Concentration difficulties
	YMRS	Elevated mood
	YMRS	Increased motor activity
	YMRS	Speech
	YMRS	Language
	YMRS	Content
Factor 3: sleep disturbance (7.5%)	MADRS	Reduced sleep
	YMRS	Sleep
Factor 4: judgment/impulsivity (6.7%)	YMRS	Sexual interest
	YMRS	Appearance
	YMRS	Insight
Factor 5: irritability/hostility (5.8%)	YMRS	Irritability
	YMRS	Disruptive, aggressive behavior

All items with factor loading ≥0.4 are shown.

MADRS, Montgomery-Åsberg Depression Rating Scale; YMRS, Young Mania Rating Scale.

### Treatment efficacy

Treatment efficacy was assessed for each treatment (aripiprazole, haloperidol, lithium, and placebo) at baseline, days 4, 7, and 10, and then weekly throughout the study period to reflect clinical decision points for potential treatment change during an acute episode. In the 12-week, active-controlled trials, efficacy was also assessed at day 2 (Table 1). The primary efficacy endpoint was mean change from baseline to study end in YMRS total score for all studies, excluding one 12-week study where the primary endpoint was treatment response, defined as ≥50% improvement from baseline in YMRS total score (Vieta et al. 2005). For the purpose of the current analysis, data from all treated subjects were pooled and standardized factor scores from principal component analysis (as described previously (Swann et al. 2013b)) converted to rating-scale-related scores. The factor conversion was performed by using the subjects who comprised the five factor scores as the basis to calculate the mean change from baseline to week 3 by factor score. The rating scale values (YMRS and MADRS items) at baseline and week 3 were used to run the analyses of covariance (ANCOVA) for mean change from baseline to week 3 by factor for each of the treatments included in these studies, with double-blind treatment and study as main effects, and baseline assessment as covariate. Factor definition is presented in Table 2. All analyses were conducted using the SAS 8.2 statistical analysis program and the last observation carried forward (LOCF).

### Predictive value of early efficacy

Efficacy data were also used to evaluate the ability of early (day 4 and week 1) improvement in factor scores to predict efficacy at endpoint (week 3), using receiver operating characteristic (ROC) curves for percentage change in factor score at day 4 and week 1 in predicting factor response at week 3. Optimal percentage cut-off scores were determined by visual inspection of the ROC curves. Factor response was defined as a 50% improvement (reduction) in factor score from baseline to week 3. Receiver operating characteristic curves were constructed through the use of logistics regression models for each of the three treatment groups: aripiprazole treatment, comparative treatment (haloperidol and lithium combined), and placebo.

The area under the ROC curve (AUC) represents the percentage of randomly drawn pairs from the early improvement time point (day 4/week 1) and endpoint (week 3) that were correctly classified, i.e., where early efficacy was predictive of endpoint efficacy. An AUC of 0.5 would suggest that the test correctly classifies these subjects only 50% of the time, while an AUC of 1 would represent correct classification 100% of the time. Additionally, the ROC curves could be used to identify the optimal percentage change in factor score at day 4/week 1 that best predicts response at week 3, by locating the point on the curve nearest to the perfect classification point of 100% (0 on the x-axis of specificity [false positive rate] and 1 on the y-axis of sensitivity [true positive rate]).

## Results

### Subjects

Of the 2,179 subjects included in the factor analysis, 2,174 subjects had baseline data and at least one post-baseline efficacy evaluation for inclusion in the efficacy sample. Subjects were assessed following treatment with aripiprazole (*n* = 1,001), haloperidol (*n* = 324), lithium (*n* = 155), and placebo (*n* = 694). Treatment continuation varied from 31% to 71% at week 3 of the original studies (El Mallakh et al. 2010; Keck et al. 2003; Keck et al. 2009; Sachs et al. 2006b; Vieta et al. 2005; Young et al. 2009).

### Treatment effect on factor score

**Figure 1:**
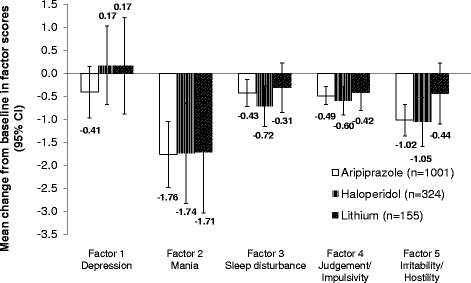
Effects of treatment (versus placebo) on factor scores from baseline to week 3 of treatment*. *Displayed as treatment difference, which is a pairwise comparison of active treatment versus placebo. CI, confidence interval.

**Figure 2:**
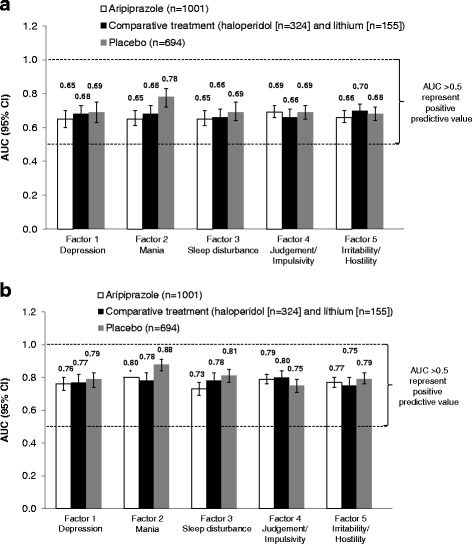
**Area under the ROC curve.** Area under the receiver operating characteristic curve for percent change in factor score at Day 4 **(a)** and week 1 **(b)** predicting factor response at week 3. *Confidence interval data not available. AUC, area under the receiver operating characteristic curve; CI, confidence interval.

The effects of treatment on factor scores are shown in Figure 1. Aripiprazole, haloperidol, and lithium significantly improved mania factor scores compared with placebo as indicated by confidence intervals. Improvements between baseline and week 3 in sleep disturbance, judgment/impulsivity, and irritability/hostility factor scores significantly favored aripiprazole and haloperidol treatments compared with placebo, while no significant improvements were observed for lithium compared with placebo (apart from factor 4). Changes in depression factor scores demonstrated the smallest treatment effect across the treatment arms and compared with placebo. Improvements in depression factor scores versus placebo showed a trend for improvement for aripiprazole at week 3 (study endpoint), but the changes did not reach statistical significance (Figure 2).

### Predictive value of early efficacy

The AUCs were >0.5 across all factors with all treatments for efficacy at day 4 and week 1 as predictors of response at week 3 (Figure 2). In general, AUCs were greater for week 1 than for day 4. The greatest AUCs for each treatment group observed at day 4 were factor 4 (judgment and impulsivity) for subjects treated with aripiprazole (0.69), factor 5 (irritability/hostility) for subjects receiving haloperidol and lithium (0.70), and factor 2 (mania) for placebo (0.78) (Figure 2). The greatest AUCs for each treatment group observed at week 1 were factor 2 (mania) for subjects treated with aripiprazole or placebo (0.80 and 0.88, respectively) and factor 4 (judgment and impulsivity) for subjects treated with haloperidol and lithium (0.80) (Figure 2). The optimal percentage improvement in factor score at day 4/week 1 for predicting factor response at week 3 was generally approximately 40% to 50% across all factors and treatments (data not shown).

## Discussion

In the current analysis, all active treatments (aripiprazole, haloperidol, and lithium) were significantly more effective than placebo for the manic factor (characterized by the classic symptoms of mania, including elevated mood and increased motor activity). A depressed mood factor was shown previously to be a key component of manic or mixed episodes associated with bipolar I disorder in subjects with acute manic or mixed episodes (Swann et al. 2013b). In the current analysis, which corrected for baseline values, the effect of treatment on depressed mood was lower than that seen with other factors. When assessing the depression mood factor, aripiprazole showed an improvement, while subjects in the haloperidol and lithium groups showed worsening compared with placebo. Although none of the changes were significant, the lack of benefit with lithium and higher effectiveness of aripiprazole compared with haloperidol at lowering depressive symptoms is in agreement with previous findings (Swann et al. 2002; Vieta et al. 2005). Bearing in mind the low level of depression at baseline, no meaningful clinical changes were expected and no definitive conclusions can be drawn from the findings presented here with regard to differential efficacy between the treatments.

Interestingly, the irritability/hostility factor - a prominent component of manic episodes - was improved with both aripiprazole and haloperidol compared with placebo. Lithium did not demonstrate improvements in the irritability/hostility factor in this study, raising the question of whether irritability/hostility factor symptoms may differentially respond to therapy. However, a relatively small effect of lithium and a significant effect of valproate on irritability/hostility have been reported previously (Swann et al. 2002).

The predictive value of response at week 3 was shown to be nearly as good at day 4 as at week 1. Early identification of treatment response or non-response in bipolar disorder may be beneficial to subjects to optimize clinical outcome by allowing identification of subjects that are most likely to substantially benefit from therapy. Similar analyses assessing benefits of early response in subjects with bipolar depression who received aripiprazole showed that the absence of minimal treatment response early during treatment predicted ultimate non-response at study endpoint with high predictive validity (Kemp et al. 2010; Kemp et al. 2011). This is broadly consistent with earlier research treatment using composite mania scores which showed that response to lithium could be predicted at day 7 (Swann et al. 1986).

It has also been shown in subjects with schizophrenia that early treatment response encourages subjects to remain on the treatment longer with a lower rate of treatment discontinuation (Kinon et al. 2008) potentially leading to improved subject functioning and a reduction in overall healthcare costs for these subjects (Ascher-Svanum et al. 2008). Therefore, identifying patients who may not respond to and/or remit on a chosen treatment could be a powerful tool that enables clinicians to formulate treatment regimens that shorten the exposure to ineffective agents or refine the dose of treatments to enhance response. Benefits of early response on the predictive value of treatment response to aripiprazole have been documented for other indications including schizophrenia and major depressive disorder (Correll et al. 2013; Muzina et al. 2011).

In the current study, overlapping confidence intervals suggest similarities in predictive power between treatments; however, no formal statistical testing was performed to directly compare the AUCs between treatments. The predictive value of the placebo response (particularly to factor 2 - mania) at both day 4 and week 1 was notably high. This is perhaps not surprising given that most of the studies (Keck et al. 2003; Keck et al. 2009; Sachs et al. 2006b; Young et al. 2009) included in this analysis required inpatient hospitalization for the first 2 weeks of treatment. Manic subjects responding to placebo at day 4 are more likely to show a sustainable placebo response at Week 3. Symptom improvement in response to placebo could have been enhanced by patients potentially displaying a transient episode of mania or the structured hospital and destimulated environment that may have shortened the natural 6- to 12-week duration of a manic episode (Keck et al. 2000; Vieta et al. 2005), as well as high frequency of control visits and the fixed-dose design of the treatment. Analysis of the ROC curves suggests that, in order to achieve a clinically relevant prediction of treatment outcome at week 3, a higher percentage cut-off than the typically used 30% was required. Therefore, the optimal early percentage improvement in factor scores is approximately 40% to approximately 50% from baseline. However, the above observation requires further analyses on large sets of data to establish a clinically meaningful threshold and potentially inform new classifications and sequential treatment strategies for bipolar disorder.

The Research Domain Criteria (RDoC) project (http://nimh.nih.gov/research-priorities/rdoc/index.shtml) has been launched by the National Institute of Mental Health (NIMH) to aid in the development and implementation of new ways of classifying psychopathology and grouping participants in research studies based on dimensions of observable behavior and neurobiological measures. The aim of the project is to create a foundational research literature that informs future versions of nosologies based upon genetics, biomarkers, and behavioral neuroscience. As an initial step, a sufficient research foundation is needed that can eventually inform the best approaches for clinical diagnosis and treatment. Factor analyses such as those presented here, while only using principles of the RDoC, allow for a dimensional approach to the examination of symptoms and associated treatment outcomes that might become the basis for comparisons of outcomes across diagnoses using data from trials that used categorical diagnoses for study entry. The current analysis provides an example of a potential dimensional structure for episodes of bipolar disorder. Factors and factor clusters developed in this manner provide a platform for neurobiological and treatment studies to support research into alternative approaches for assessing psychopathology based on more basic characteristics that may underlie symptomatic presentation of bipolar disorder.

Although the study above was not designed to directly compare treatment outcome between different agents, it may provide a promising construct for future studies to investigate the potential for symptom factors to characterize the effects of treatment on patients with acute mania and to assess the value of early efficacy in predicting treatment outcome. Clinicians and patients are still using the DSM for diagnosis, and the construct of mania is not fully explained by any domain currently in the RDoC. However, data presented here may be relevant to provide both patients and clinicians with the tools to make a more informed decision on short-term and inevitably long-term treatment to maximize patient benefit.

### Limitations

This *post hoc* analysis includes the pooled data of several clinical trials. While this is a limitation, it should be noted that the majority of the studies included (El Mallakh et al. 2010; Keck et al. 2003; Keck et al. 2009; Sachs et al. 2006b; Young et al. 2009) used relatively similar study inclusion criteria and thus resulted in similar subject populations suitable for pooling. However, it should also be considered that the inclusion criteria of the original trials may limit the generalizability of the study findings. The 12-week studies included in this analysis were not powered to detect differences between active treatments at week 3. Subject numbers for haloperidol and lithium were substantially lower than for aripiprazole and placebo and may have limited the power for detecting relationships involving these treatments. Another limitation is the use of LOCF data in the context of investigating symptom change; however, regarding the short duration of these studies and the relatively high completion rate (up to 71% at week 3), LOCF data was deemed suitable to assess efficacy at the indicated time points. The rating scale values (YMRS and MADRS items) at baseline and week 3 were used to run the ANCOVA for mean change from baseline to week 3 by factor for each of the three treatments with double-blind treatment and study as main effects and baseline assessment as covariate. A factor conversion was performed rather than presenting endpoint data as raw score and adjusting for baseline response, or providing the change in score and regressing it on treatment.

## Conclusions

This *post hoc* analysis of data from over 2,000 subjects with manic or mixed episodes associated with bipolar I disorder, enrolled in six double-blind, randomized, controlled clinical trials, confirmed that all active treatments provided significant improvement compared with placebo in at least one factor (mania), with aripiprazole and haloperidol resulting in significant efficacy in four out of the five factors assessed. Early efficacy was found to be predictive of efficacy at week 3 for all treatments across all factors; however, response at week 1 was a better early predictor than response at day 4. This analysis confirms the value of early treatment/assessment across a range of factors from this heterogeneous subject population and may be useful as a means for examining symptom dimensions in treatment trials across diagnoses. While exploratory, results such as these might be part of future risk prediction models across diagnoses - including characteristics not collected in a clinical trial setting, potential biomarkers, and genetic markers - that might lead to a more precise means of picking treatments and predicting response for our patients.

## Abbreviations

ANCOVA: analysis of covariance
AUC: area under the ROC curve
DSM-V: Diagnostic and Statistical Manual of Mental Disorders, Fifth Edition
FDA: Food and Drug Administration
LOCF: last observation carried forward
MADRS: Montgomery-Åsberg Depression Rating Scale
NIMH: National Institute of Mental Health
RDoC: The Research Domain Criteria
ROC: receiver operating characteristics
YMRS: Young Mania Rating Scale
